# Regenerative repair is connected to early and specific structural, immune, and metabolic MSC signatures in adult mammals

**DOI:** 10.1038/s41598-026-58327-y

**Published:** 2026-06-19

**Authors:** Miguel Thomas, Anastasia Pacary, Emmanuelle Arnaud, David Bernard, Cathy Maugis-Rabusseau, Christophe Guissard, Adèle Arlat, Noélie Davezac, Anne Lorsignol, Jenny Paupert, Elsa Gratianne Guillot, Arnaud Bonnaffoux, Béatrice Cousin, Valérie Planat-Benard, Paul Monsarrat, Louis Casteilla, Marielle Ousset

**Affiliations:** 1 University of Toulouse, CNRS UMR 5070, INSERM U1301, EFS, ENVT, Institut RESTORE, Toulouse, France; 2 Univ Toulouse, INSA Toulouse, CNRS, IMT, Toulouse, France; 3 Oral Medicine Department and CHU de Toulouse, Toulouse Institute of Oral Medicine and Science, Toulouse, France; 4 Cancer Research Center of Lyon, Computational Biology Division, Léon Bérard Center, INSERM U1052-CNRS9, UMR5286, Claude Bernard Lyon1 University, Lyon, France; 5 Synergie Lyon Cancer Foundation, Lyon, France; 6 Artificial and Natural Intelligence Toulouse Institute ANITI, Université de Toulouse, Toulouse, France

**Keywords:** Tissue regeneration, Mesenchymal stromal cells (MSCs), Cellular heterogeneity, scRNA-seq, SIM, Biotechnology, Cell biology, Stem cells

## Abstract

Adult mammals exhibit a limited capacity for tissue regeneration following injury and typically heal through scar formation. Mesenchymal stem/stroma cells (MSCs), which are phenotypically plastic and ubiquitous across tissues, play a critical role in maintaining tissue architecture during repair. We hypothesized that early events in regenerative and non-regenerative repair involve changes in MSC heterogeneity, which in turn determine repair outcomes. To test this hypothesis, we performed extensive single-cell RNA sequencing (scRNA-seq) in a mouse model of tissue injury. This model standardizes the comparison of non-regenerative and regenerative repair in adults with identical developmental stages and genetic backgrounds. Our analysis of MSCs during the early phases of tissue repair in adult mammals enabled the identification of distinct regenerative and non-regenerative MSC clusters, suggesting that specific MSC states may actively drive tissue repair outcomes. Furthermore, unsupervised approaches allowed us to revisit the functional signatures of MSCs centering on their impact on tissue structure (S), inflammation/immunity (I) and metabolism (M). By integrating these S, I and M functions, the SIM framework provides a conceptual model to interpret MSC behavior as a coordinated tissue-level response rather than a collection of isolated pleiotropic activities. This work positions MSCs at the center of the “SIM” triad, underscoring their pivotal role in tissue repair.

**Supplementary Information:**

The online version contains supplementary material available at https://doi.org/10.1038/s41598-026-58327-y.

## Introduction

In contrast to lower vertebrates or neonatal mammals, adult mammals exhibit a limited capacity for tissue regeneration - the complete recovery of architecture and function of organs following an injury or disease. Instead tissue repair in adult mammals favors scarring pathways, characterized by excess extra-cellular matrix (ECM) deposition that can lead to organ dysfunction^1^. To advance our understanding of tissue repair and develop new therapeutic strategies, the identification of cell populations associated with regenerative processes is essential.

Recently, it has been proposed that healthy tissue function is achieved and maintained through an optimized relationship between three transversal multiscale elements: the structure/supportive compartment (S), the immune system (I) and metabolism (M), collectively referred to as the SIM paradigm^2–4^. The S compartment serves as the source and the driver of tissue architecture and repair^5^. Tissue integrity is maintained through appropriate relationships between tissue architecture and its functions, resulting from constant, bidirectional interactions between two cellular compartments: the parenchyma and the stroma. While the parenchymal compartment, composed of differentiated cells and their precursors/progenitors/stem cells, is specific to the physiological functions of the tissue, the supporting stromal compartment (primarily comprising hematopoietic, endothelial, and mesenchymal stem/stroma cells or MSCs) represents a cross-sectional element throughout the body that plays a central role in tissue architecture and repair^5^. The immune/inflammatory system (I) is critical for signaling repair and defending the body’s integrity after injury, ensuring the maintenance of healthy architecture and functioning. Immune cells are transiently recruited and activated to manage the body’s response to various injuries, and the orchestration of innate and adaptive immune cell subpopulations determines the outcome of repair. Energy metabolism (M) is essential for maintaining cellular and tissue architecture and healthy function, regardless of environmental variations. An organism’s ability to respond effectively to daily stress relies on the coordinated action of the three elements of the SIM triad: support tissues, which guide the reconstruction of tissue architecture; the immune/inflammatory system, which provides adequate alerting, continuous monitoring, and signals for process resolution; and metabolism, the engine of energy supply. The SIM paradigm emerges as a central pathophysiological framework for understanding the mechanisms of tissue regeneration. Indeed, the “SIM” triad, where these three components coexist and act synergistically, is transversal at all biological levels, from the organism as a whole, to the tissue and cellular levels^2,4^.

MSCs are localized throughout all the tissues and are key actors in homeostasis and repair processes^2,6^. Based on investigations of MSCs, including in clinical trials^7^, MSCs are classically defined as multipotent cells capable of differentiating into mesenchymal lineages (osteoblasts, chondrocytes, tenocytes, adipocytes and myofibroblasts) and exhibit extensive paracrine activity including supportive and immunosuppressive features^8,9^. From a functional perspective, MSCs also play a pivotal role in conditioning tissue and whole-organ architecture through their ability to secrete and remodel the ECM, which shapes the mechanical macrostructure of tissues. This function occurs in coordination with endothelial and immune cells as well as parenchymal cells. Rather than acting through isolated pleiotropic effects such as ECM remodeling^10^, immunomodulation^11^, or trophic support, angiogenesis^12^, MSCs are increasingly recognized as tissue-coordinating stromal cells. They integrate mechanical, inflammatory, and metabolic cues to orchestrate local repair responses^13^. Within the SIM paradigm^2–4^, MSCs could function as dynamic sensing-and-response cells capable of regulating multicellular interactions within the tissue microenvironment. In doing so, they influence immune responses, progenitor cell activation, vascular remodeling, and fibrotic outcomes depending on the pathological context.

MSCs exhibit high heterogeneity, not only across tissues but also within the same tissue, in both humans and mice^14,15^. Adipose-derived stromal cells (ASCs), the resident MSC population of adipose tissue (AT), similarly display substantial phenotypic heterogeneity^16–18^. While this diversity has been extensively characterized in non-injured conditions, the existence of distinct regenerative versus fibrotic MSC subpopulations emerging after tissue injury remains less explored. Nevertheless, accumulating evidence suggests the existence of pre-existing or injury-induced profibrotic MSC subpopulations in several pathological contexts. For example, CD9^high^ MSCs in obesity-induced AT fibrosis and ADAM12^+^ MSCs following muscle and dermal injuries suggest that a fraction of MSCs may already be committed toward a fibrotic phenotype before injury^19,20^. Despite their heterogeneity, MSCs’ high phenotypic plasticity and their involvement in all steps of repair processes, their role in the early steps of tissue repair remains under-investigated. We hypothesized that the loss of regenerative capacity in adult mammals is related to an early qualitative change in MSC heterogeneity.

To test this hypothesis, we utilized an original and standardized mouse model of tissue injury that enables the comparison of non-regenerative and regenerative processes of the same tissue in adult mammals with identical genetic backgrounds and developmental stage. This model involves performing calibrated subcutaneous AT (scAT) injury in adult C57BL/6 mice. We previously demonstrated that regeneration can be restored in spontaneously scarring mice through transient pharmacological treatment with Naloxone (NalM), an opioid antagonist, administered immediately after injury^21–23^. Tissue regeneration was assessed using a regeneration index, defined as the weight ratio between the resected scAT and the uninjured contralateral scAT. Additionally, regeneration was evaluated based on the restoration of tissue architecture, characterized by the re-establishment of lobular adipocyte organization surrounded by collagen fibers and vascular networks^21,23^. The anti-regenerative role of opioids was further validated in the gold-standard regeneration model, the zebrafish^21^, as well as in pancreas regeneration^24^, underscoring the relevance of our model for identifying pro-regenerative cues conserved across species and organs. Mechanistically, we showed that the effect of NalM was related to the regulation of the inflammatory phase^22^.

In this study, we provided a comprehensive single-cell RNA sequencing (scRNA-seq) analysis of MSCs, during the early steps of regenerative and non-regenerative tissue repair in adult mammals. This analysis investigates MSC heterogeneity and revisits the functional definition of MSCs through the SIM framework.

## Results

### scRNA-seq of scAT ASCs reveals sweeping transcriptomic changes early after injury

**Fig. 1:**
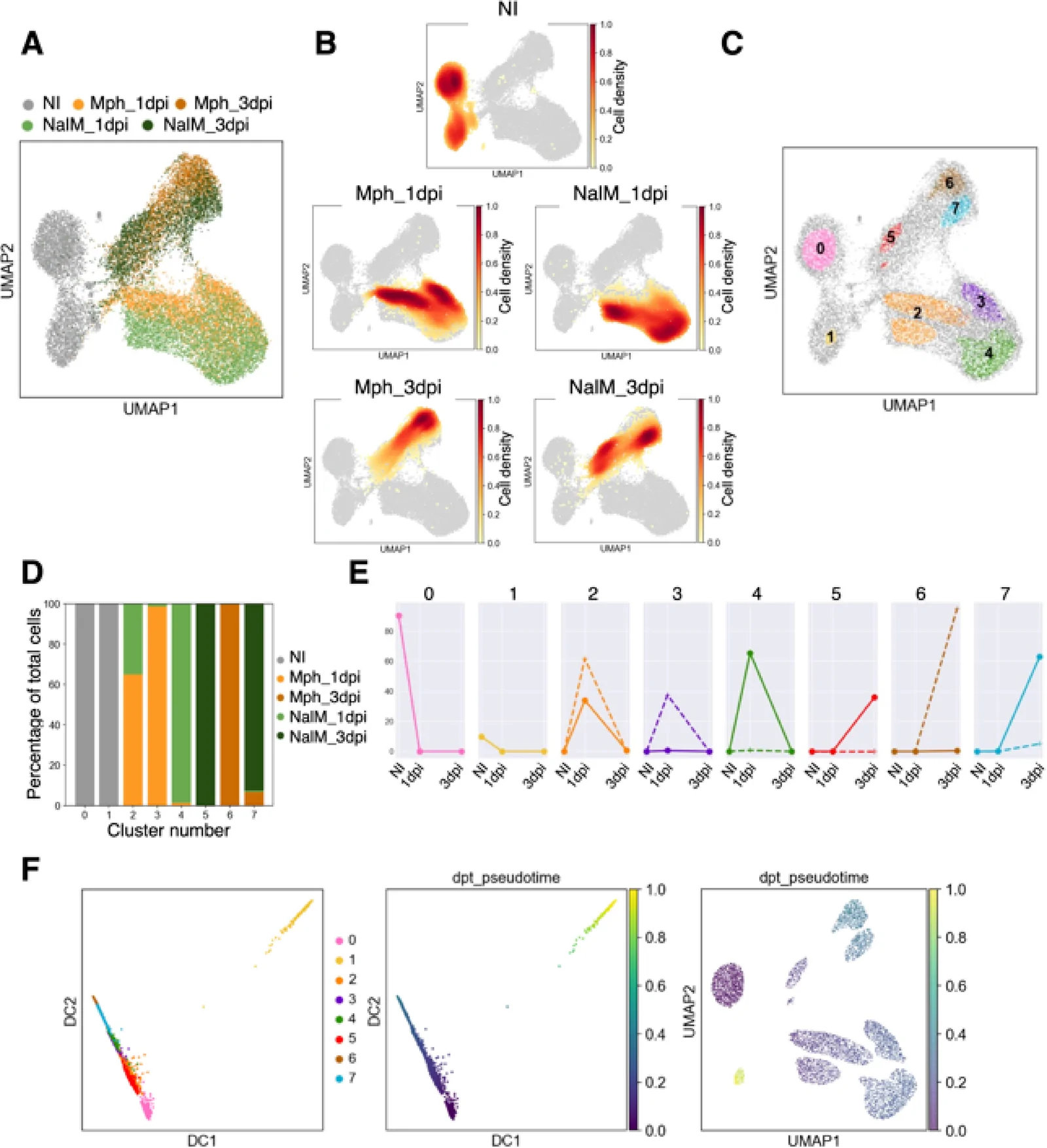
scRNA-seq reveals ASC clusters enriched with cells associated with regenerative or non-regenerative conditions. SVF cells were isolated from 3–5 pooled male C57BL/6J adult mice (6–7 weeks old), from the area corresponding to the further injured area in non-injured (NI) conditions, and from the reconstructed area 1 and 3 days after scAT injury (1dpi and 3 dpi) upon regenerative (NalM) and non-regenerative (Mph) conditions. The merged data set contains 19,595 ASCs. (**A**) UMAP colored per conditions. (**B**) UMAPs representing cell densities per condition. (**C**) Agglomerative clustering on cells selected on UMAP densities revealed 8 distinct cell clusters. Grey cells were not assigned to a cluster. (**D**) Percentage of cells from each condition per cluster. (**E**) Cell population percentages according to experimental conditions (solid lines corresponding to regenerative (NalM) or NI conditions (for cluster 0 and 1), and dashed lines corresponding to non-regenerative (Mph) conditions). (**F**) Pseudotemporal cell ordering of clusters 0 to 7 along differentiation trajectories using dpt from Scanpy, for which interstitial progenitor cluster (cluster 0) was defined as the starting one. Right panel represents the pseudotemporal coloration on UMAP.

The CD45-CD31- population contains 85% of native ASCs^25^. To assess the cellular and molecular heterogeneity of ASCs, we performed scRNA-seq, one and three-days (1dpi and 3 dpi) after scAT injury on CD45 and CD31 double negative sorted cells, thus excluding hematopoietic and endothelial cells, respectively. We merged CD45-CD31- cells from all data sets corresponding to non-injured (NI), non-regenerative (Mph 1dpi and 3dpi) and regenerative (NalM 1dpi and 3 dpi) conditions and first removed low-quality cells. We then performed a UMAP dimension reduction on an aggregate of 19,595 ASCs (Fig. 1A), which revealed different isolated groups (NI, 1 and 3 dpi) with the two clusters corresponding to the NI condition clearly distinct from two branches corresponding to 1 and 3 dpi respectively. This suggests a rapid and sudden shift of the molecular signature in ASCs from NI tissue from 1 dpi.

To characterize the different ASC populations in NI conditions, we performed an agglomerative clustering (Supplementary Fig. 1A, B). Previously published studies have already described two main clusters of ASCs within scAT: “interstitial progenitors” and “committed preadipocytes”^16–18^. Based on their marker genes, we identified that cluster 0 (pink), specifically expressing *Dpp4*, *Wnt2*, *Bmp7*, *Pi16*, *Creb5*,* Ebf2*, and cluster 1 (yellow) specifically expressing *Icam1*, *Pparg*, *Fabp4*, *Cd36*, *Col4a1*, *Col6a2*, corresponded to “interstitial progenitors” and “committed preadipocytes” populations respectively (Supplementary Fig. 1C). Interestingly, cluster 0 also expressed canonical markers of ASCs, *Cd34 and Ly6a* (Sca1), whereas these markers were down regulated in “cluster 1 (Supplementary Fig. 1D). A differentially expressed gene (DEG) analysis between these two clusters suggested that both populations primarily exhibited ECM structuration and remodeling capacities with specific changes in genes related to collagen, proteoglycans, cross-linking factors and degradation enzymes (Supplementary Fig. 1E). Additionally, we identified a third less dense population expressing tissue factor F3 (*Cd142*) as well as *Fmo2*, *Meox2*, *Vit* and *Gdf10*, which was previously described by Merrick et al.^18^ (Supplementary Fig. 1C).

### scRNA-seq reveals ASC clusters enriched with cells associated with regenerative or non-regenerative conditions

Unlike Leiden clustering that did not allow us to identify clusters specifically enriched for our different experimental conditions (Supplementary Fig. 2), agglomerative clustering of ASCs in 1 and 3 dpi branches identified 8 distinct clusters of ASCs (Fig. 1B, C) enriched with cells isolated either in non-injured (clusters 0 and 1), non-regenerative (clusters 3, 6) or regenerative (clusters 4, 5, 7) conditions. Finally, one mixed cluster (cluster 2) was present in the 1 dpi branch (Fig. 1C–E). Interestingly, by performing pseudo-temporal cell ordering on the clustering from Fig. 1C, using cluster 0 as the starting point, we identified a cell continuum that excluded cluster 1 (Fig. 1F). This suggested that ASCs, with transcriptomic profile altered by tissue injury, originated from “interstitial progenitors” and that tissue injury did not mobilize adipose-lineage-committed ASCs in the earliest steps of tissue repair. To avoid introducing noise into the data, we excluded cluster 1 from subsequent analyses.

### Tissue regeneration is associated with ASC clusters exhibiting specific balance between tissue structure (S), immune (I), and metabolic (M) responses

**Fig. 2:**
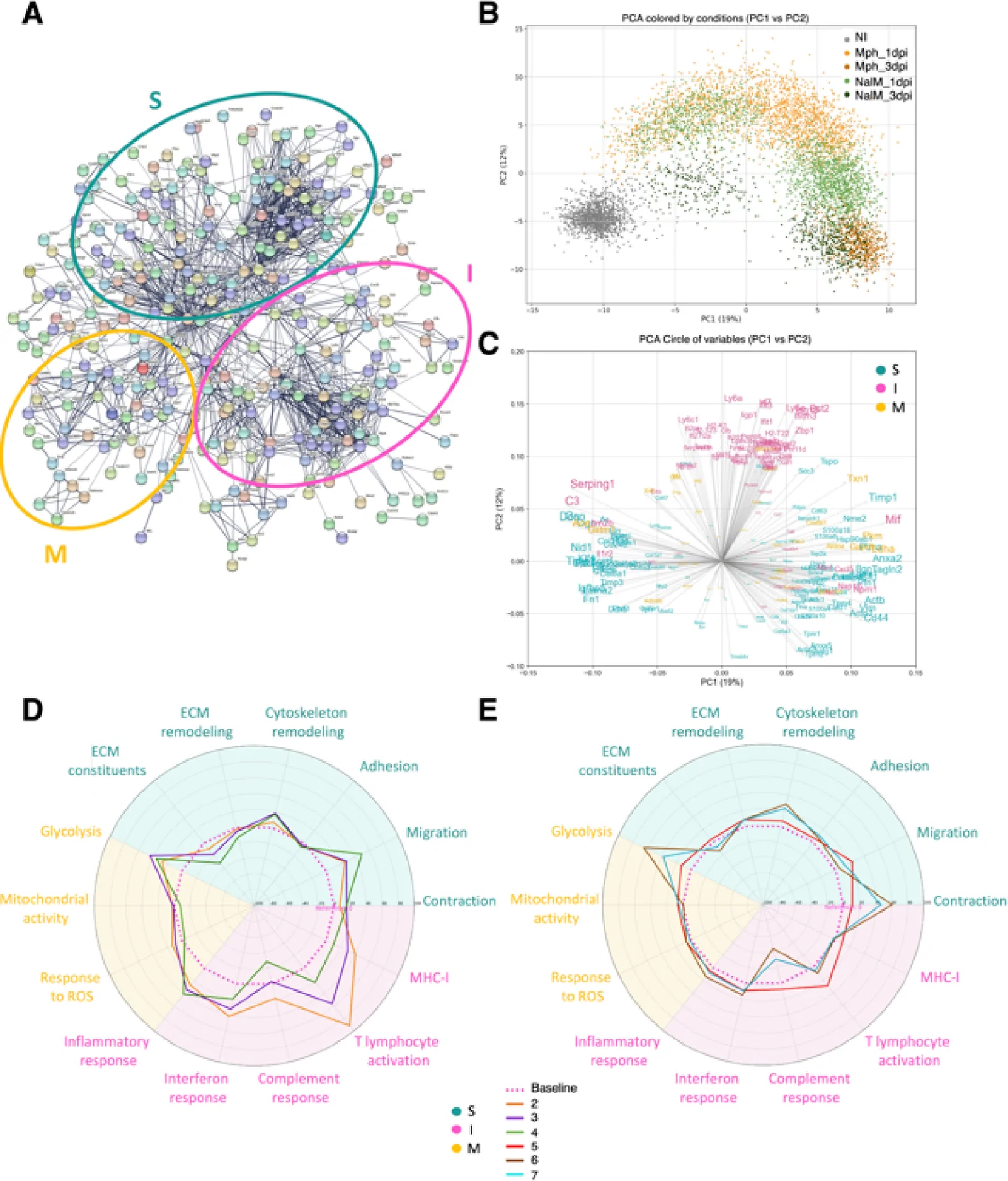
(**A**) The lists of top 100 DEGs per cluster were merged and analyzed using STRING (minimum required interaction score: 0.6). Three main gene clusters were identified and classified into structural (S), immune (I) and metabolic (M) functions. (**B**) PCA plot representing PC1 and PC2 colored by conditions. (**C**) PCA circle of variables plot representing PC1 and PC2 colored by S, I and M functions. (**D**, **E**) Radar plots using SIM functional subgroups as axes. The mean values of the z-scores for all genes belonging to the SIM subgroups per cluster were used as a metric to illustrate the integrated SIM response of ASCs 1 dpi (**D**) and 3 dpi (**E**). Each z-score was normalized using the NI condition i.e. cluster 0 “interstitial progenitors” as baseline.

To analyze the differences between ASC clusters, we performed differentially expressed genes (DEG) analyses, focusing on the top 100 DEGs per cluster. Out of the 700 DEGs identified, approximately half were redundant. After combining the gene lists, we obtained a final list of 374 genes and performed a qualitative analysis using STRING, to classify our transcriptomic data according to protein activities. We generated a functional protein interaction network based on physical and functional interactions (Fig. 2A). This network revealed 3 main groups of genes/proteins which could be classified in an unsupervised manner into structural (S), immune (I) and metabolic (M) functions. These groups included 252 out of 374 genes, (67% of DEGs), while the remaining genes were either disconnected nodes or associated with basic cellular functions, such as transcription, translation, protein folding, and secretion. Next, we performed k-means gene clustering associated with a Gene Ontology (GO) analysis using STRING. This analysis identified functional subgroups of genes within the three main SIM categories: ECM constituents, ECM remodeling, cytoskeleton remodeling, adhesion, contraction and migration for S function, interferon response, inflammatory response, T lymphocyte activation, complement response, major histocompatibility complex I (MHC-I) for I function, and glycolysis, mitochondrial activity, response to reactive oxygen species (ROS) for M function (Fig. 2A and Supplementary Fig. 3). Altogether, these functions summarize the SIM response of ASCs during tissue repair. In a second step, to determine whether DEGs related to our SIM analysis could differentiate between experimental conditions and cluster cells, we performed a principal component analysis (PCA). Three first components of the PCA capture 39% of the variance (Supplementary Fig. 4A). PCA contributions revealed that PC1, which distinguish between NI and injured ASCs (Fig. 2B, Supplementary Fig. 4B), was primarily explained by S-function-related-genes, with opposing contribution from ECM constituents and cytoskeleton remodeling genes (Fig. 2C and Supplementary Fig. 4C). PC2 and PC3 explained differences based on experimental conditions and the days post-injury (Fig. 2B and Supplementary Fig. 4B, D, E) and were associated with I-function and M-function gene expression respectively (Fig. 2C and Supplementary Fig. 4F). This qualitative analysis confirmed that the list of DEGs aligns with the SIM framework and provided insight into the SIM phenotype of ASCs involved in tissue repair.

To further understand the changes in the complex ASC phenotypes, we constructed radar plots for each ASC cluster (Fig. 2D, E) and per condition (Supplementary Fig. 5). These plots used the SIM functional subgroups as axes, with values expressed as z-score for comparability. Each score was normalized using the NI condition (i.e. cluster 0 “interstitial progenitors”) as baseline. 1 dpi associated clusters were primarily characterized by an increase in the expression of genes related to glycolysis, migration and inflammatory/immune responses except for the complement axis which showed overexpression only in non-regenerative conditions compared to the baseline (Supplementary Fig. 5). 3 dpi associated clusters were mainly characterized by the overexpression of genes related to contraction, cytoskeleton remodeling and glycolysis, alongside a down-regulation of migration-related genes compared to 1 dpi ones (Supplementary Fig. 5). Notably, the overexpression of contraction- and glycolysis-related genes was significantly higher in non-regenerative conditions compared to regenerative ones (Supplementary Fig. 5).

The SIM profiles of individual clusters 1 dpi revealed that clusters 3 and 4 (specific to non-regenerative and regenerative conditions, respectively) displayed similar global SIM profiles with higher expression of genes related to cytoskeleton remodeling, migration, inflammatory/immune response (except for complement response) and glycolysis (Fig. 2D). Notably, the SIM axes of cluster 4 were consistently lower than those of cluster 3 except for the migration axis (Fig. 2D). Cluster 2 (mixed, including ASCs from both regenerative and non-regenerative conditions) exhibited a SIM profile similar to cluster 3 and 4 with a higher expression of inflammatory/immune response-related genes (Fig. 2D). In contrast to 1 dpi clusters, the SIM analysis of 3 dpi individual clusters revealed that cluster 5 exhibited a distinct global SIM profile compared to other 3 dpi clusters, with uniformly distributed but higher z-scores for all SIM axes relative to the baseline (Fig. 2E). Clusters 6 and 7 were highly similar, showing specific overexpression of genes related to cytoskeleton remodeling, contraction and glycolysis, alongside downregulation of complement response-related genes (Fig. 2E). Taken together, these results revealed subtle but significant differences in SIM profiles for ASCs associated with regenerative or non-regenerative conditions. These differences suggest that the early steps of tissue repair are associated with distinct clusters exhibiting specific SIM signatures which may determine the final repair outcome.

### scRNA-seq reveals two functionally distinct ASC populations, *Cd34 + Dpp4+* and *Cd34-Dpp4-*, during tissue repair

**Fig. 3:**
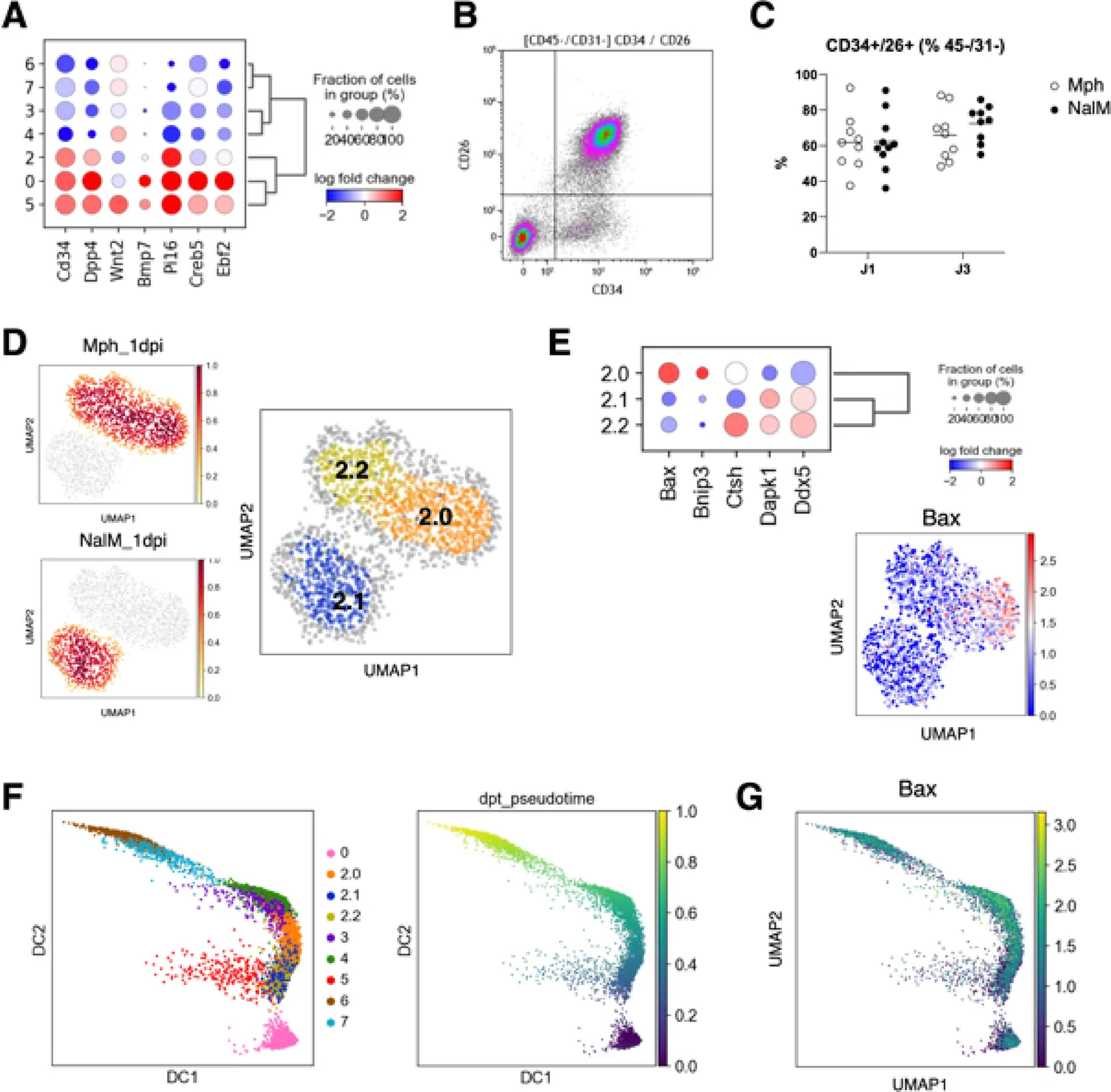
scRNA-seq reveals two functionally distinct ASC populations, *Cd34 + Dpp4+* and *CD34-Dpp4-*, during tissue repair. (**A**) Dot plots representing at the same time Fold change (FC) expression levels of selected genes (color map) and the fraction of cells expressing these genes among cell clusters (circle size) for marker genes of ‘interstitial progenitors’ identified in NI conditions. (**B**) Representative dot plot of flow cytometry showing the expression of CD34 and CD26 among singlet, alive, CD45-CD31- cells. (**C**) Percentage of CD34 + CD26+ among CD45-CD31- cells and quantified by flow cytometry in regenerative and non-regenerative conditions 1 and 3 dpi. (**D**) UMAPs representing cell densities per condition for cluster 2. Agglomerative clustering on cells selected on UMAP densities revealed 3 distinct cell sub-clusters. Grey cells were not assigned to a cluster. (**E**) Dot plots representing at the same time Fold change (FC) expression levels of selected genes (color map) and the fraction of cells expressing these genes among cell clusters (circle size) for cell death associated genes and individual gene UMAP distribution of the pro-apototic gene *Bax*. (**F**) Pseudotemporal cell ordering without cluster 1 of clusters 0 to 7 along differentiation trajectories using dpt from Scanpy, for which interstitial progenitor cluster (cluster 0) was defined as the starting one. (**G**) Pseudotemporal cell ordering colored with *Bax* gene expression.

To deeply understand the cellular hierarchies governing regenerative and non-regenerative pathways, we performed hierarchical clustering based on gene expression (Supplementary Fig. 6A). The “interstitial progenitors” gene signature was monitored over time after injury in both the 1 dpi and the 3 dpi branches. The dendrogram identified two distinct cluster groups: one overexpressing interstitial progenitor-associated genes (clusters 0, 2, and 5) and one downregulating these genes (clusters 3, 4, 6, and 7) (Fig. 3A). Post-injury, clusters 2 and 5 were closest to cluster 0, and expressed *Cd34* and *Dpp4* genes (Fig. 3A). Flow cytometry experiments validated the expression of the surface markers CD34 and CD26 (*Dpp4*) at the protein level (Fig. 3B), and confirmed the existence of two main populations based on these markers: CD34 + CD26+ and CD34-CD26-, with similar proportions between regenerative and non-regenerative conditions (Fig. 3C). Clusters 2 and 5 also overexpressed the same marker genes and exhibited similar ECM structuration (expression of collagens and other ECM components genes) and remodeling capacities (expression of degradation and cross-linking genes). In contrast, these genes were down-regulated in all other post-injury clusters (clusters 3, 4, 6, 7) (Fig. 3A, Supplementary Fig. 6B). Cluster 4 (regenerative, 1 dpi) specifically expressed genes involved in remodeling (*Adam12* and *Mmp14*) and cross-linking (*Lox*), while lacking gene expression linked to ECM structuration (collagen expression) (Supplementary Fig. 6B). In contrast, at 3 dpi, clusters 6 (non-regenerative) and 7 (regenerative) appeared to recover the expression of genes related to ECM structuration and slightly increased the expression of those related to remodeling capacities (*Adam12* and *Mmp14*) primarily under regenerative conditions (cluster 7) (Supplementary Fig. 6B). Clusters 0, 2 and 5 featured a lower proliferation signature compared to clusters 3, 4, 6 and 7 (Supplementary Fig. 6C). These observations align with the fact that proliferative ASCs lose CD34 expression over time^25^ and during tissue repair (Supplementary Fig. 6D).

To deepen our understanding of the cellular hierarchies governing regenerative and non-regenerative pathways, we focused on *Cd34 + Dpp4+* ASCs and sub-clustered the mixed cluster (cluster 2) corresponding to ASCs at 1dpi. This analysis revealed 3 distinct sub-clusters (Fig. 3D). Clusters 2.0 and 2.2 were enriched with cells isolated in non-regenerative conditions, in contrast to cluster 2.1 that was enriched with cells isolated in regenerative conditions. Differential expression analysis revealed that in cluster 2.0, genes related to cell death (*Bax* and *Bnip3*) were upregulated, while anti-apoptotic genes (*Ctsh*, *Dapk1*, *Ddx5*) were down-regulated (Fig. 3E). The comparison between cluster 2.1 and 2.2 revealed that cluster 2.2 over-expressed genes associated with ECM components (collagens, proteoglycans) whereas cluster 2.1 exhibited a higher expression of transcription factors (e.g. *Cebp*, *Junb*) (Supplementary Fig. 6E). Overall, these data suggest that a subset of *Cd34 + Dpp4+* ASCs associated with non-regenerative conditions undergoes apoptosis at 1 dpi, while the remaining cells are involved in ECM structuration. Under regenerative conditions, *Cd34 + Dpp4+* ASCs undergo a reactivation of their transcriptional program, potentially paving the way for tissue regeneration. To further refine this analysis, we performed pseudo-temporal cell ordering excluding cluster 1 and using cluster 0 as the starting point, along with sub-clustering of cluster 2. This approach revealed two distinct cellular trajectories (Fig. 3F). Both trajectories originated from a common node composed of sub-clusters 2.1 and 2.2. One trajectory extended toward a continuum linking the apoptotic sub-cluster 2.0 with clusters 3, 4, 6, and 7, reflecting a degree of cellular selection (Fig. 3G). The other trajectory lead toward cluster 5. These findings suggest a direct relationship between sub-cluster 2.1 (1 dpi) and cluster 5 (3 dpi), both of which are enriched in cells isolated under regenerative conditions.

## Discussion

For investigating regenerative processes, most studies primarily focus on the inflammatory phase and immune/inflammatory cells, and touch on subsequent phases such as ECM reconstruction and stem cell differentiation, or compare regenerative and non-regenerative conditions across different genetic backgrounds, developmental stages, or tissues^26–29^. In contrast, our work specifically investigated MSC heterogeneity in the earliest steps of tissue repair, within the same tissue and under identical genetic and developmental conditions. We demonstrated that shifts in MSC functional profiles occur at very early stages following injury, highlighting the originality of this study in identifying these early changes that support tissue regeneration during the initial phases of tissue repair in adult mice.

In-depth scRNA-seq analysis revealed a complex and dynamic transcriptomic landscape reflecting MSC heterogeneity. The description and evolution of this landscape provide a new understanding of MSCs through three functional components: their role in tissue structuring (S), their immunomodulation functions (I), and their ability to regulate metabolic flows (M). MSCs appear to be key players at the center of the “SIM” triad at the cellular level, and our results highlight the relevance of the SIM framework as a tool to define the functional significance of MSCs in various contexts. Notably, the SIM framework emerged from unsupervised approaches, independently converging toward a SIM-like interpretation of MSC states. This spontaneous emergence strongly reinforces the biological relevance and robustness of the SIM framework as an integrative model for understanding MSC functions during tissue repair. The SIM framework provides a broad, integrative readout of cellular states, capturing the complexity of MSC functional diversity. A key advantage of the GO analysis performed with STRING is its ability to identify gene subgroups within the three major SIM categories (S, I, and M). These subgroups (6 for S, 5 for I and 3 for M) are visually represented in the radar plots. At 1 dpi, qualitative analysis of the radar plots revealed that the different populations (clusters 2, 3 and 4) vary in the same overall direction, though quantitative differences are observed across each of the GO-defined subgroups. At 3 dpi, similar trends were noted for clusters 6 and 7, but the analysis also highlighted the emergence of a distinct cluster (cluster 5) associated with regeneration, which exhibits a clearly distinct SIM profile. These observations suggest that MSC functional changes arise from the cumulative effect of coordinated modulations across multiple biological processes. Unlike the classical cell-centered view - describing MSCs through isolated, pleiotropic functions^10–12^- the SIM framework offers a physiological and tissue-level functional definition of MSCs. To further refine our understanding, we propose that defining a composite “SIM index” integrating these summed functional modifications, could provide deeper insights into MSC functions and improve the prediction of their therapeutic effects. Overall, our analysis underscores both the complexity and the subtle functional differences between MSC populations associated with regenerative and non-regenerative conditions. These findings, combined with a previous study reporting age-related changes in gene expression at the tissue level in human gingiva^3^, corroborate that the SIM viewpoint is transversal to tissue and cell levels^2,4^.

Our study revealed that tissue repair after injury relies on distinct MSC clusters, enabling us to distinguish major and minor features of tissue regeneration versus non-regenerative conditions. Among the major features, we identified a *Cd34 + Dpp4+* cluster specific to regenerative conditions at 3 dpi, exhibiting a unique SIM signature. Unlike other MSC clusters identified during the repair process - which prioritize one or two SIM functions - this cluster displays an uniformly distributed balance of SIM functions comparable to that of native ‘interstitial progenitors’ but with higher z-scores. This suggests that a *Cd34+* /*Dpp4 +* MSC/ASC subset with this SIM signature may be necessary for regeneration. However, it remains to be determined whether the presence of this subset al.one is sufficient to enable regeneration. It would also be important to test whether cultured ASCs used in therapy display the SIM regenerative profile, given that CD34 expression significantly decreases during in vitro proliferation and expansion prior to therapeutic use^25^.

Another key feature was the identification of a cluster overexpressing apoptotic genes at 1 dpi under non-regenerative conditions, suggesting a specific cell selection process at this stage. A similar mechanism may occur under regenerative conditions but with distinct timing. Evidence is accumulating that oxidative stress drives cellular responses post-injury, inducing massive, non-specific cell death that clears the repair area^5^. Our study suggests that this selection process is not wide but targeted toward specific cell clusters. Further investigation is required to understand why and how apoptosis is critical for committing cells toward a regenerative repair outcome. Additionally, our study revealed that the I (immune) component may predominate during the early steps of tissue repair, particularly at 1 dpi. The importance of the I response of MSCs is consistent with a recent report describing structural cells as key regulators of the organ immune response^30^. Among the elements identified within the I component, the differential expression of genes related to complement pathways is intriguing and underscores the key but neglected role of complement activation kinetics during repair in mammals^31^. This importance has already been demonstrated in gold-standard regeneration models, such as blastema formation in axolotl limb regeneration^32^. Parallel to these major observations, our SIM analysis allowed us to decipher subtle mechanisms, relying on distinct but closely related MSC clusters, between regenerative and non-regenerative conditions. A regenerative cluster at 1 dpi exhibited slight differences compared to other clusters, characterized by a SIM signature with a slightly lower I gene and a highest migration signature, involving an increase in Podoplanin (*Pdpn)* gene expression. This suggests rapid inflammatory-induced tissue MSC remodeling at this stage. These results, combined with previous research in reindeer, strongly suggests that specific fibroblast-immune cell interactions drive the regenerative pathway^26^. These observations are also consistent with the increase in Podoplanin expression in fibro*-*adipogenic progenitors at 0.5 dpi in skeletal muscle regeneration^33^. Moreover, non-regenerative conditions were characterized by a slight increase in the contraction axis score at 3dpi, consistent with the observation that an absence of contraction is associated with regeneration in mouse^34^.

The lack of a cellular continuum between the different time points (NI, 1 and 3 dpi) and the fact that early tissue repair did not mobilize adipose-lineage-committed ASCs - as seen in our pseudotime analysis - suggest a rapid switch in cell phenotypes during the early phase of repair. This underscores the need for further studies incorporating earlier and later time points to better understand MSC dynamics and cell interactions, as well as the underlying gene regulatory networks driving the rapid phenotypic shift associated with tissue regeneration.

While our findings may support broader inter-species and inter-organ generalization, several limitations should be acknowledged: (i) this study was performed in young mice and focused on one MSC-rich tissue, which may limit direct extrapolation to other physiological or pathological contexts; (ii) our analysis relied exclusively on transcriptomic data and did not capture the full cellular complexity of the tissue; (iii) tissue dissociation required for scRNA-seq may selectively alter or eliminate rare and fragile MSC subpopulations; and (iv) the absence of spatial information currently limits the interpretation of local cellular interactions. Future studies will therefore be required to validate these newly identified MSC subpopulations at the functional level and to confirm whether their proposed SIM profiles and derived composite “SIM index” reliably predict regenerative or therapeutic potential. Because the SIM framework provides a physiological and tissue-level perspective of MSC biology, rather than a purely cell-centered view based on isolated functions, this may offer a more relevant framework for therapeutic stratification and prediction of MSC efficacy, particularly given the inherently pleiotropic nature of these cells. Supporting this hypothesis, recent studies have demonstrated that a physiological, tissue-level perspective can better predict therapeutic effects than conventional cell-centered approaches^3,35^. In particular, the SIM framework has enabled the identification of molecular signatures and markers associated with human health status in cultured skin fibroblasts, suggesting that integrated SIM-related profiles may capture biologically and clinically relevant functional states that are not detectable through classical phenotypic characterization alone.

Altogether, the fine deciphering of MSC heterogeneity and dynamics through the SIM analysis suggests a rapid, complex, heterogeneous and crucial involvement of MSCs very early in repair processes that cannot be explained by the expression of few markers alone. Further investigations will be required to validate the regenerative potential after engraftment of selected clusters and to identify putative regenerative master transcription factors responsible for these dynamics.

## Conclusion

Our study demonstrates that regeneration relies on rapid and dynamic shifts within heterogeneous MSC subpopulations. This highlights the central regulatory role of MSCs during the earliest steps of tissue repair. By integrating structural, immune, and metabolic functions, the SIM framework provides a conceptual model to interpret MSC behavior and its change as a coordinated tissue-level response rather than isolated pleiotropic activities. The identification of distinct regenerative and non-regenerative MSC clusters suggests that specific MSC states may actively drive tissue repair outcomes. These findings open new therapeutic perspectives based on the selection, enrichment, or reprogramming of MSC subpopulations exhibiting regenerative SIM profiles while limiting profibrotic or non-regenerative states. Altogether, our work supports the development of functional and context-dependent MSC-based therapies guided by integrated SIM signatures rather than by conventional marker expression alone.

## Methods

### Animals

Experiments were performed on 5–7 week-old male C57BL/6 mice (Envigo, Gannat, France). Animals were group-housed in a controlled environment (12-h light/dark cycles at 21 °C) with unrestricted access to water and a standard chow diet and bred in a conventional facility at the Faculty of Pharmacy in Toulouse. Before tissue removal, mice were killed by cervical dislocation.

### Ethics declarations

All animal experiments were carried out in compliance with European Community Guidelines (2010/63/UE), and approved by the Ethics Committee in Animal Experimentation (C2EA 122 Toulouse) and by the institutional review board of the Ministry of Higher Education and Research (MESR) under the protocol number #40880-2023010612201954 v6.

### Sub-cutaneous AT (scAT) injury and treatments

Animals were anesthetized by inhalation of isoflurane (2.5%) and subjected to a single incision on the abdomen to access and resect 35–40% of the right scAT between lymph node and groin. The skin was then closed with three suture points. Mice were then injected subcutaneously at the surgery site, once a day from d0 to d3 post-injury with NalM (17 mg/kg, Sigma Aldrich, Saint Louis, MO, USA) or Mph (subcutaneous injection, 10 mg/kg, Francopia, Antony, France) to induce regenerative or non-regenerative outcomes respectively, as previously described^21^. Although endogenous opioids inhibit regeneration in adult control mice, treatment with Morphine (Mph) reduces inter-individual variability in the regenerative response. This observation supports our decision to use Mph-treated mice for the non-regenerative group in this study.

### Isolation of adipose-derived stromal vascular cell fraction (SVF)

Mice were sacrificed by cervical dislocation in NI conditions and 1- and 3-days post-injury in regenerative or non-regenerative conditions. For injured conditions, we considered the reconstructed tissue at the lesion margin, from which a 2 mm-wide strip adjacent to the lesion edge was collected. For controls, the corresponding tissue region in non-injured mice was used. Tissues sustained a mechanical dissociation and enzymatic digestion at 37 °C with collagenase (NB4, Sigma-Aldrich, Saint-Louis, USA) for 30 min. Cells from the scAT SVF were collected by centrifugation after elimination of undigested fragments by filtration as previously described^36^. Red blood cells were removed by incubation in hemolysis buffer (140 mM NH4Cl and 20 mM Tris, pH 7.6). Cells were then counted and used for single cell isolation, cell sorting and flow cytometry.

### Flow cytometry and cell sorting analyses

Freshly isolated SVF cells were stained in PBS containing FcR-blocking reagent (BD Pharmigen, Franklin Lakes, USA) for 30 min on ice with fluorochrome-conjugated antibodies (Abs). Phenotyping was performed by immunostaining with conjugated rat anti-mouse Abs and compared with isotype-matched control Abs (Supplementary Table S1). Cell viability was assessed using Live/Dead cell viability assay (Thermo Fisher Scientific, Waltham, USA).

For cell-sorting experiments, SVF cells were stained with CD45 and CD31 antibodies. Double negative cells for these two markers were gated and sorted (BD FACSAria Fusion III Cell sorter, BD Biosciences, Franklin Lakes, USA). For flow cytometry, SVF cells were stained with CD45, CD31, CD34 and CD26 antibodies, and analyzed on an LSR Fortessa flow cytometer (BD Biosciences, Franklin Lakes, USA). Data acquisitions and analyses were performed using Diva (BD Biosciences, Franklin Lakes, USA) and Kaluza software (Beckman Coulter, Brea, USA), respectively.

### Single cell isolation, libraries preparation and scRNA-seq

Collected samples containing a mix of 1:10 CD45/CD31 double positive versus double negative cells were centrifuged at 300x g for 10 min at 4°C and resuspended in PBS. The number of cells loaded on the system was calculated based on the desired number of captured cells following manufacturer’s instructions. A volume of each suspension containing 10,000 cells was loaded in a single channel of a Chromium Single Cell Instrument (10x Genomics, Pleasanton, CA, USA), and barcoded cDNA libraries were prepared using Chromium Single Cell 3’ Reagent Kits v3.1 (10x Genomics, Pleasanton, CA, USA) according to the manufacturer’s protocol. Amplified cDNA from each channel was used to construct a sequencing library (Illumina, San Diego, CA, USA). cDNA libraries were checked for quality using HS NGS Fragment Kit on Fragment Analyser (Agilent Technologies, Santa Clara, USA) and quantified by qPCR. Sequencing was performed on a NextSeq550 System (Illumina, San Diego, CA, USA) using 2 × 75 bp sequencing to an average of 25,000 reads per cell. The scRNA-seq data reported in this publication have been deposited in the Functional Genomics Data Array Express (EMBL-EBI) with the accession number (E-MTAB-14552).

### scRNA-seq data processing

ScRNA-seq data were processed using Cell Ranger v6.1.1 (10X Genomics; RRID: SCR_017344). Each sequenced library was demultiplexed to individual cell FASTQ files utilizing the cellranger mkfastq program. The library was aligned to an indexed GRCm38 (mouse) RefSeq genome with default parameters, followed by barcode counting, and UMI counting using Cell Ranger. Downstream analysis was performed on filtered feature counts generated by Cell Ranger. Data sets analyzed in this study: (1) non-injured conditions (NI): 4,815 single cells were sequenced with 17,640 mean reads and a median of 2,520 detected genes per cell, (2) morphine treatment 1-day post-injury (Mph_1dpi): 4,498 single cells were sequenced with 20,174 mean reads and a median of 2,728 detected genes per cell, (3) morphine treatment 3-days post-injury (Mph_3dpi): 2,803 single cells were sequenced with 27,897 mean reads and a median of 3,238 detected genes per cell, (4) NalM condition 1-day post-injury (NalM_1dpi): 4,559 single cells were sequenced with 20,109 mean reads and a median of 2,244 detected genes per cell, (5) NalM condition 3-days post-injury (NalM_3dpi): 2,920 single cells were sequenced with 31,323 mean reads and a median of 3,478 detected genes per cell. 5 tissues were mixed per condition.

### Statistical analyses of scRNA-seq data

We merged all data sets or analyzed the NI data set on its side. An initial quality control filtering was performed. First, we identified potential single-cell doublets using the DoubletDetection Python package. Further low-quality single cells containing < 250 expressed genes and > 0.2% mitochondrial transcripts, as well as less than 2,000 counts and more than 50,000 counts were excluded from the analysis. Following the removal of low quality and doublet cells, bioinformatics processing of the scRNA-seq data was performed using Single Cell Analysis in Python (Scanpy) toolkit. Single cell data were normalized and log-transformed. We applied principal component analyses to reduce the dimensionality of the data. The top 100 principal components, selected using the Elbow plot method, were used in the UMAP analysis. We then performed cell clustering using agglomerative clustering on cells selected on UMAP densities (threshold 70% of density). We used the rank genes groups function to identify cluster marker genes, enrich function to perform over-representation analyses (ORA) using gene ontology (GO) biological processes and diffmap function to perform pseudotime analyses. STRING Core Data Resource (https://string-db.org) was used using a minimum required interaction score of 0.6. Differential gene expression z-scores were calculated using the Scanpy package (sc.tl.rank_genes_groups) between the different clusters. To illustrate the integrated SIM response of cells, we calculated per cell cluster or per condition for each SIM subgroup the mean values of the z-scores for the genes within this subgroup.

## Supplementary Information

Below is the link to the electronic supplementary material.

Supplementary Material 1

## Data Availability

Requests for further information and resources should be directed to and will be fulfilled by the lead contact, Marielle Ousset (marielle.ousset@inserm.fr).- The scRNA-seq data that support the findings of this study have been deposited in the Functional Genomics Data Array Express (EMBL-EBI) with the accession number E-MTAB-14552 (https:/www.ebi.ac.uk/biostudies/arrayexpress/studies/E-MTAB-14552).
