# Supplementary material for: Regenerative repair is connected to early and specific structural, immune, and metabolic MSC signatures in adult mammals
Source: Sci Rep. 2026 Jun 19;16:28244. doi: 10.1038/s41598-026-58327-y (PMC13558711; doi:10.1038/s41598-026-58327-y)
Supplement: Supplementary file 1 [file 41598_2026_58327_MOESM1_ESM.pdf]

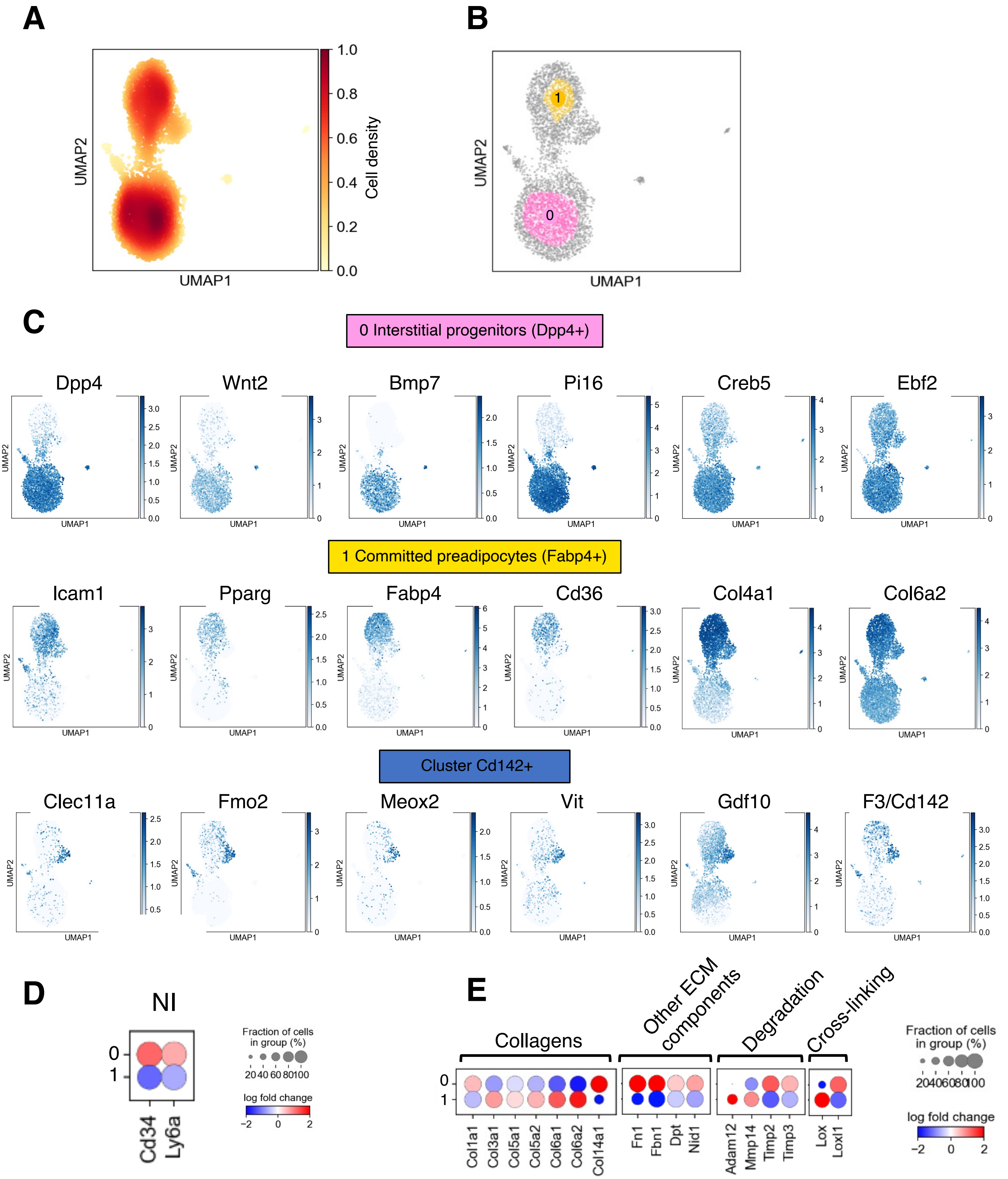

Supplementary Figure 1. Characterization of ASCs in NI conditions. (A-B) Agglomerative clustering on cells selected on UMAP densities of 4,815 NI ASCs revealed 2 distinct cell clusters represented on UMAP. Grey cells were not assigned to a cluster. **(C)** Individual gene UMAP showing the expression levels and distributions of representative marker genes. **(D)** Expression of canonical markers of ASCs (*Cd34* and *Ly6a*) upon NI conditions. Dot plots represent at the same time Fold change (FC) expression levels of selected genes (color map) and the fraction of cells expressing the selected genes among cell groups (circle size). **(E)** Dot plots representing at the same time Fold change (FC) expression levels of selected differentially expressing genes (DEGs) (color map) and the fraction of cells expressing these genes among cell clusters (circle size). Functional DEGs were selected among top 50 DEGs and classified upon ECM components, degradation and cross-linking.

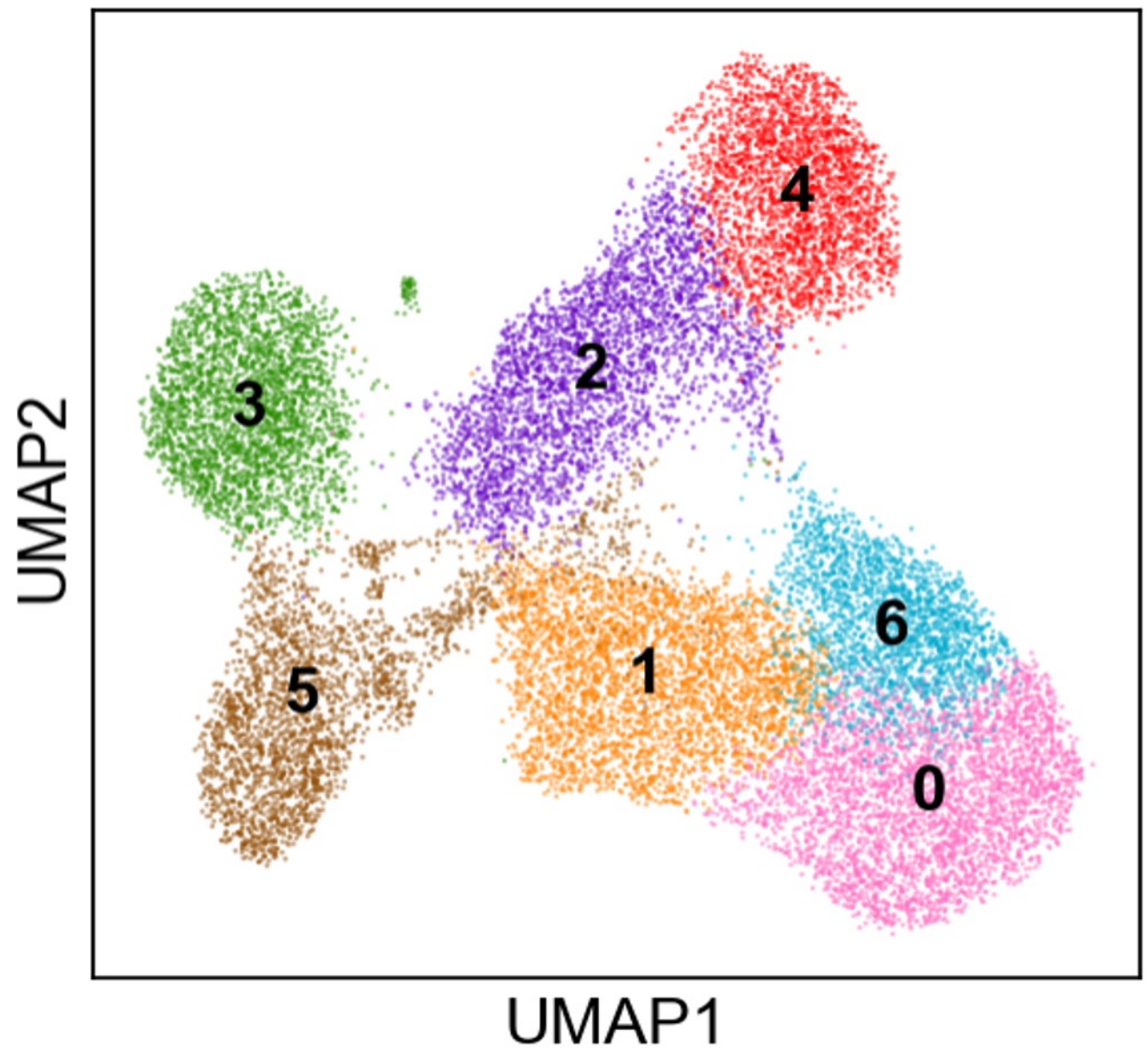

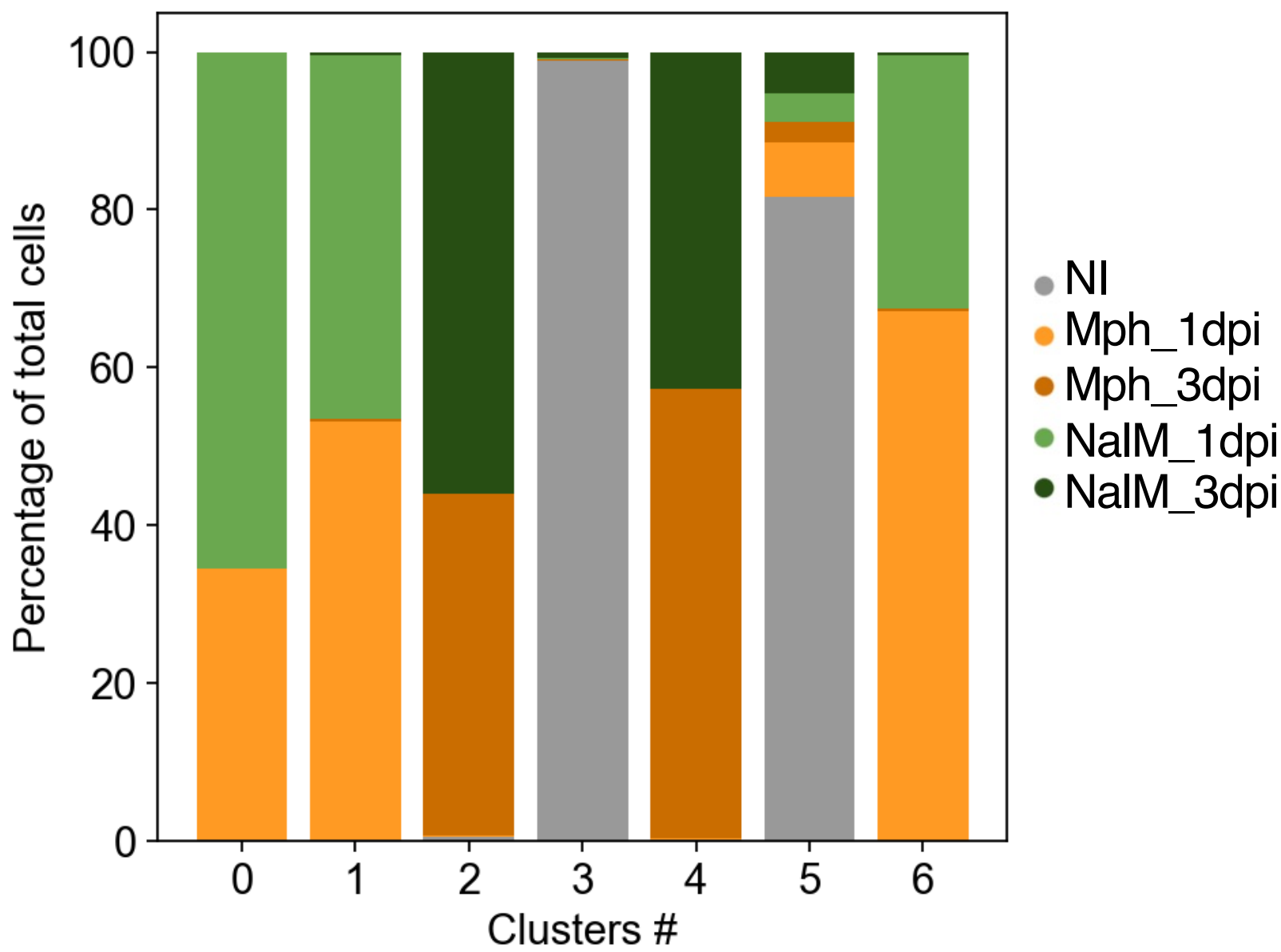

Supplementary Figure 2. Leiden clustering did not identify clusters specifically enriched for our different experimental conditions. SVF cells were isolated from 3-5 pooled male C57BL/6J adult mice (6-7 weeks old), from the area corresponding to the further injured area in non-injured (NI) conditions, and from the reconstructed area 1 and 3 days after scAT injury (1dpi and 3 dpi) upon regenerative (NaIM) and non-regenerative (Mph) conditions. The merged data set contains 19,595 ASCs. **(A)** UMAPs representing Leiden clustering. **(B)** Percentage of cells from each condition per cluster.

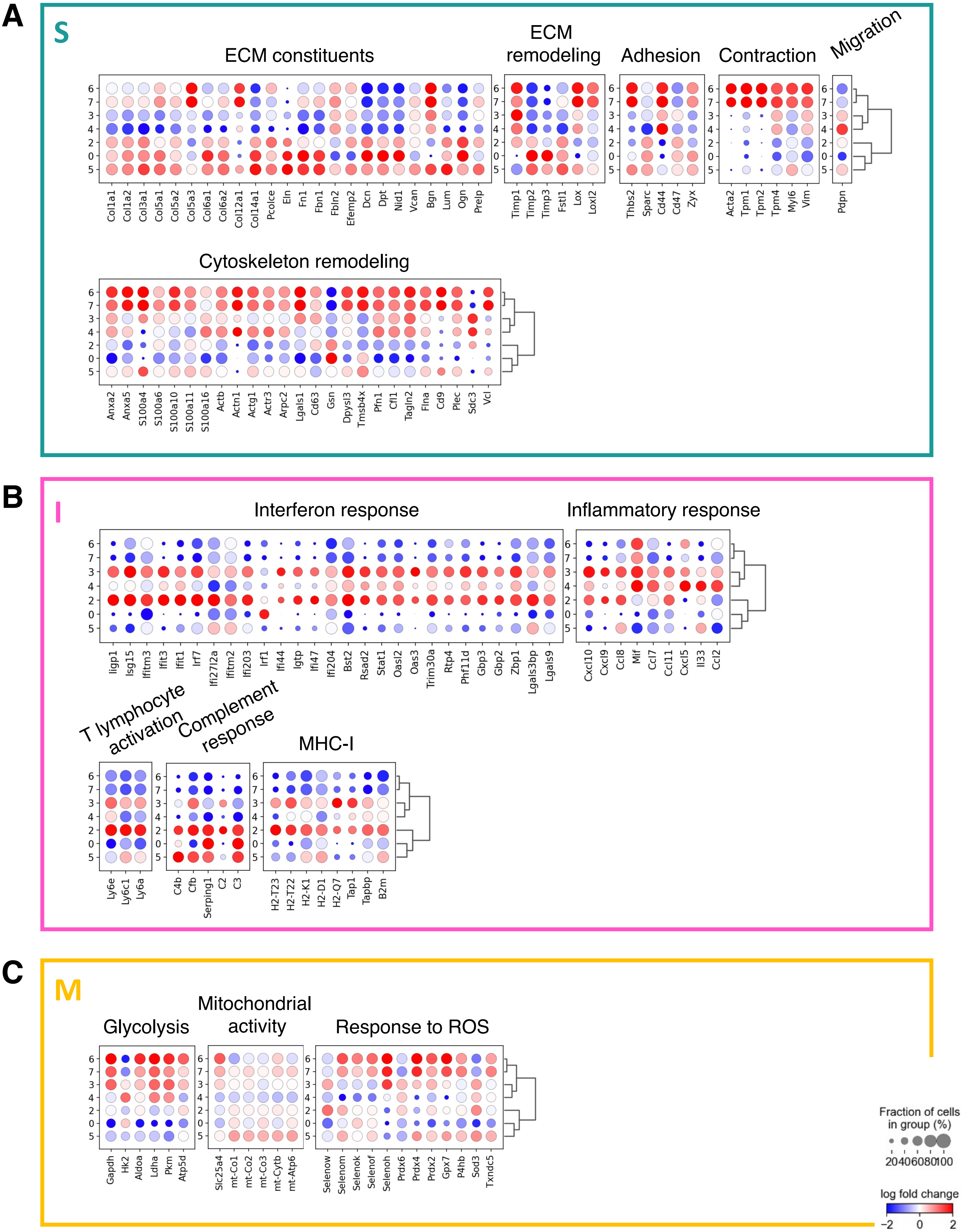

Supplementary Figure 3. Characterization of *CD34+Dpp4+* and *CD34-Dpp4-* ASCs during tissue repair according to S, I and M functions. Dot plots representing at the same time Fold change (FC) expression levels of 143 SIM related genes (color map) and the fraction of cells expressing these genes among cell clusters (circle size). Dot plots were classified according to functional sub-groups of genes per main functions S **(A)**, I **(B)** and M **(C)**.

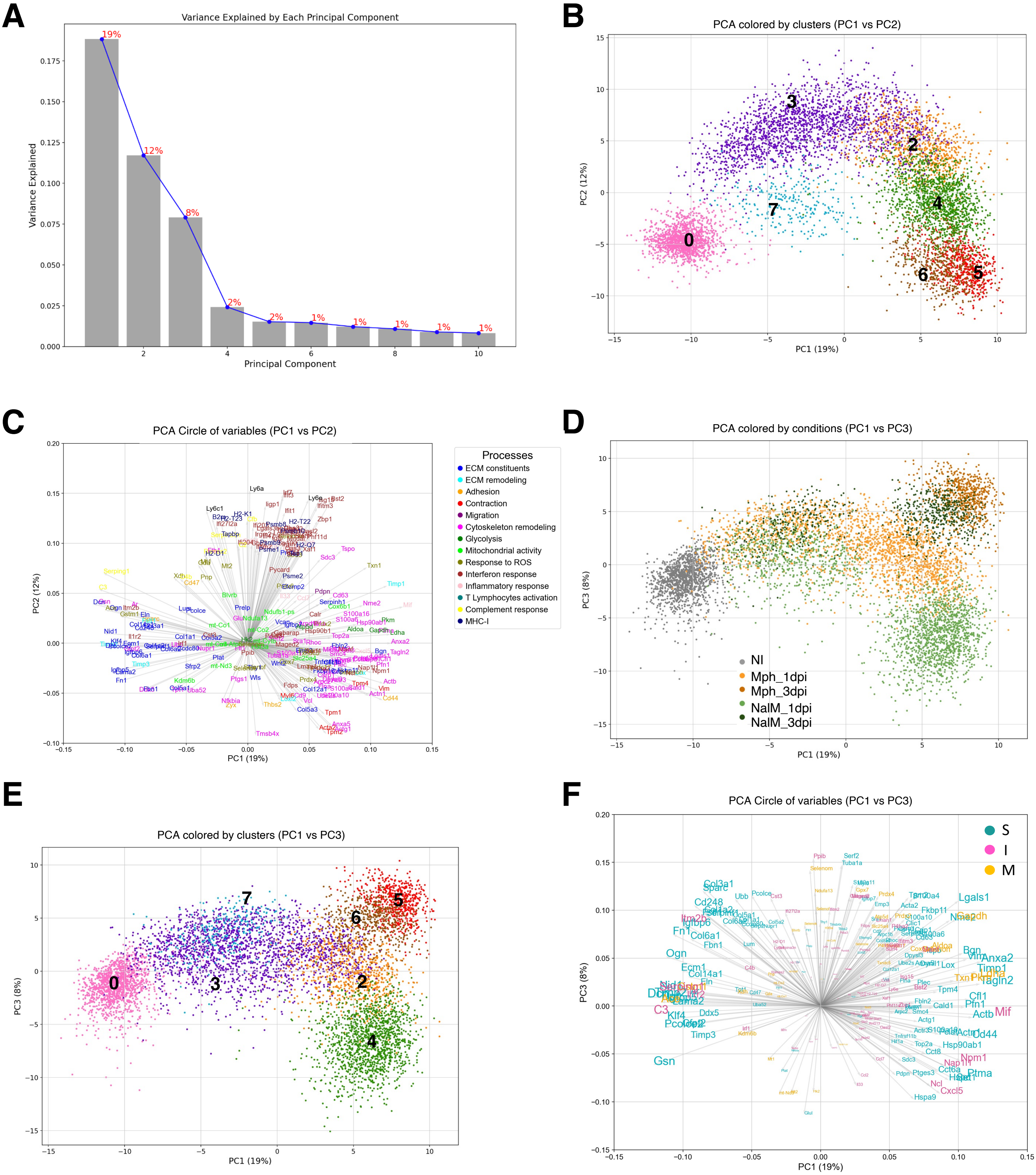

Supplementary Figure 4. PCA analysis based on the list of DEGs. (A) Scree plot showing the variance explained by each component in PCA, between our five conditions. **(B)** PCA plot representing PC1 and PC2 colored by clusters. **(C)** PCA circle of variables plot representing PC1 and PC2 colored by S, I and M sub-groups. **(D-E)** PCA plot representing PC1 and PC3 colored by conditions **(D)** or by clusters **(E)**. **(F)** PCA circle of variables plot representing PC1 and PC3 colored by S, I and M functions.

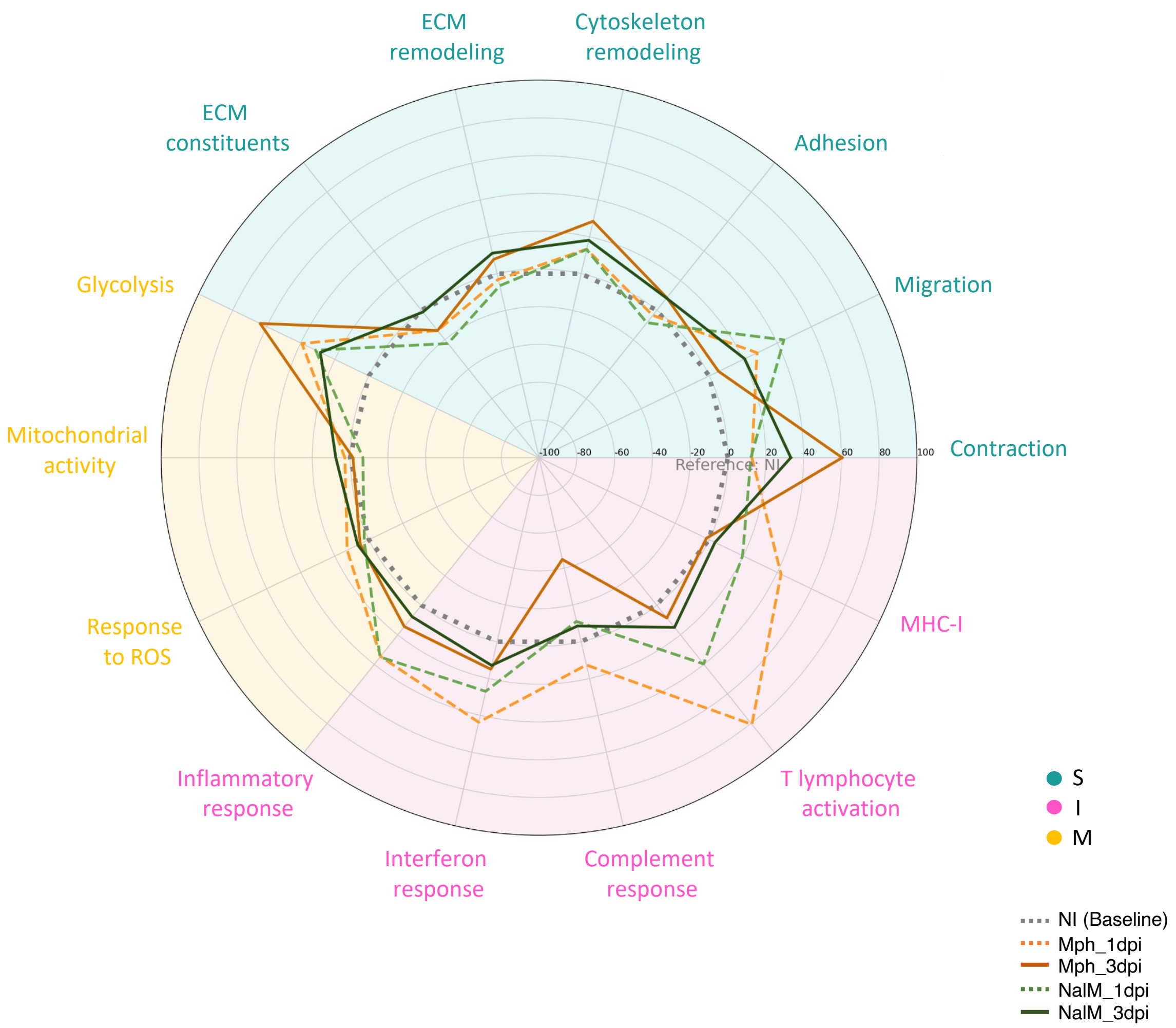

Supplementary Figure 5. Radar plots illustrating the SIM analysis of ASCs after injury. Radar plots using SIM functional sub-groups as axes. The mean values of the z-scores for all genes belonging to the SIM subgroups per condition were used as a metric to illustrate the integrated SIM response of ASCs. Each z-score was normalized using the NI condition as baseline.

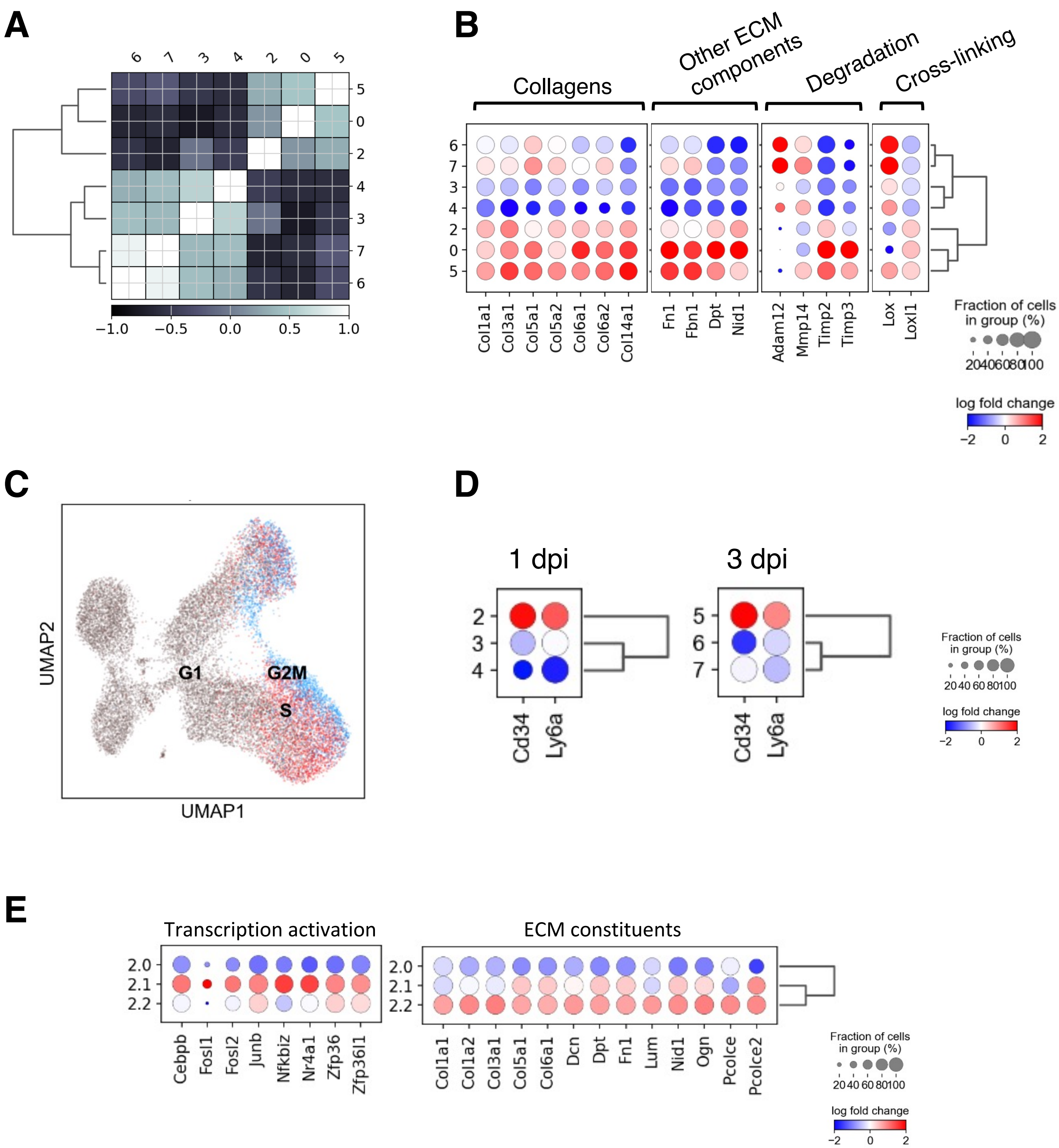

Supplementary Figure 6. Characterization of ASCs during tissue repair. (A) Correlation plot after hierarchical clustering dendrogram analysis between the average gene expression profile of clusters. **(B)** Dot plots representing at the same time Fold change (FC) expresion levels of selected genes (color map) and the fraction of cells expressing these genes among cell clusters (circle size) for functional DEGs of 'interstitial progenitors' identified in NI conditions and classified upon ECM components, degradation and cross-linking. **(C)** UMAP colored by cell cycle phases. **(D)** Expression of canonical markers of ASCs (*Cd34* and *Ly6a*) 1 and 3 dpi. Dot plots represent at the same time Fold change (FC) expression levels of selected genes (color map) and the fraction of cells expressing the selected genes among cell groups (circle size). **(E)** Dot plots representing at the same time Fold change (FC) expression levels of selected genes (color map) and the fraction of cells expressing these genes among cell clusters (circle size) for list of genes corresponding to functional DEGs.

Supplementary Table S1: Anti-mouse antibodies used for cell sorting and flow cytometry

Antibody	Clone	Manufacturer
**CD45**	30-F11	BioLegend
**CD31**	MEC13.3	BD Bioscience
**CD34**	MEC14.7	BioLegend
**CD26**	L272	BD Bioscience
